# Dramatic changes induced on porous silicon birefringence by shape-dependent properties

**DOI:** 10.1038/s41598-026-41405-6

**Published:** 2026-03-30

**Authors:** Guido Mula, Muhammad Naseem Akhtar, Francesca Assunta Pisu, Stéphane Bastide, Angelo Angelini, Mateo Rosero-Realpe, Luca Boarino

**Affiliations:** 1 PoroSiLab, Dipartimento di Fisica, Università degli Studi di Cagliari, Cittadella Universitaria di Monserrato, S.P. 8 Km 0.700, Monserrato (Ca), Italy; 2 UMR 7182, Univ Paris Est Créteil, CNRS, ICMPE, 2 Rue Henri Dunant, 94320 Thiais, France; 3 Advanced Materials & Life Sciences Division, Istituto Nazionale di Ricerca Metrologica, Strada Delle Cacce 91, 10135 Turin, Italy; 4 Department of Applied Science and Technology (DISAT), Politecnico Di Torino, C.So Duca degli Abruzzi 24, 10129 Torino, Italy

**Keywords:** Porous silicon, Form birefringence, Porosity, Materials science, Optics and photonics, Physics

## Abstract

Porous silicon birefringence originating from preferred pore etching directions along the crystallographic axes has been reported in a number of scientific publications along the last three decades. This birefringence originates from the fact that the preferred pore etching directions are those along the main crystallographic axes, namely [100], [010] and [001]. The preferred surface orientation for the studies on birefringent porous Si is [110], where the main optical axis is horizontal and, for a light beam propagating along the direction perpendicular to the surface, there are two distinct refractive indexes, so that this configuration has been considered easier for actual devices fabrication and is, by large, the most studied as far as birefringence is concerned. In this work, centered on (100)-oriented porous silicon, we show that the nature of porous silicon birefringence depends on the porous structure configuration and is therefore also dependent on the orientation of the surface of the bulk Si used. Moreover, we demonstrate that such structural dependence is so large that even apparently similar configurations can lead to positive or negative birefringence. Possible reasons for these remarkable differences are discussed along the experimental results.

**Supplementary Information:**

The online version contains supplementary material available at 10.1038/s41598-026-41405-6.

## Introduction

Porous silicon (PSi) is a very interesting and versatile material. It has been discovered by Uhlir^1,2^ in the 1950s and gained considerable interest when its photoluminescence in the visible region was discovered by Canham^3^. Its high specific surface^4,5^ and versatility have made this material of interest for a wide range of possible purposes. Applications of PSi spans from biosensors^6–9^, to MicroElectroMechanical Systems (MEMS)^10^, to drug delivery systems^11–14^, to optical devices^15–22^. The fabrication of organic/inorganic hybrids with PSi has also been widely investigated^23–30^. An intense research effort has been devoted to the understanding of PSi properties and on the control of its fabrication processes^31–33^. Although the most diffused method for the fabrication of PSi is electrochemistry (EC)^31,33,34^, that is electrochemical corrosion of doped bulk Si, many other methods as Metal-Assisted Chemical Etching^35–39^ have been investigated, along completely different strategies as the production from plants^40^ or from porous silica^41^.

**Fig. 1:**
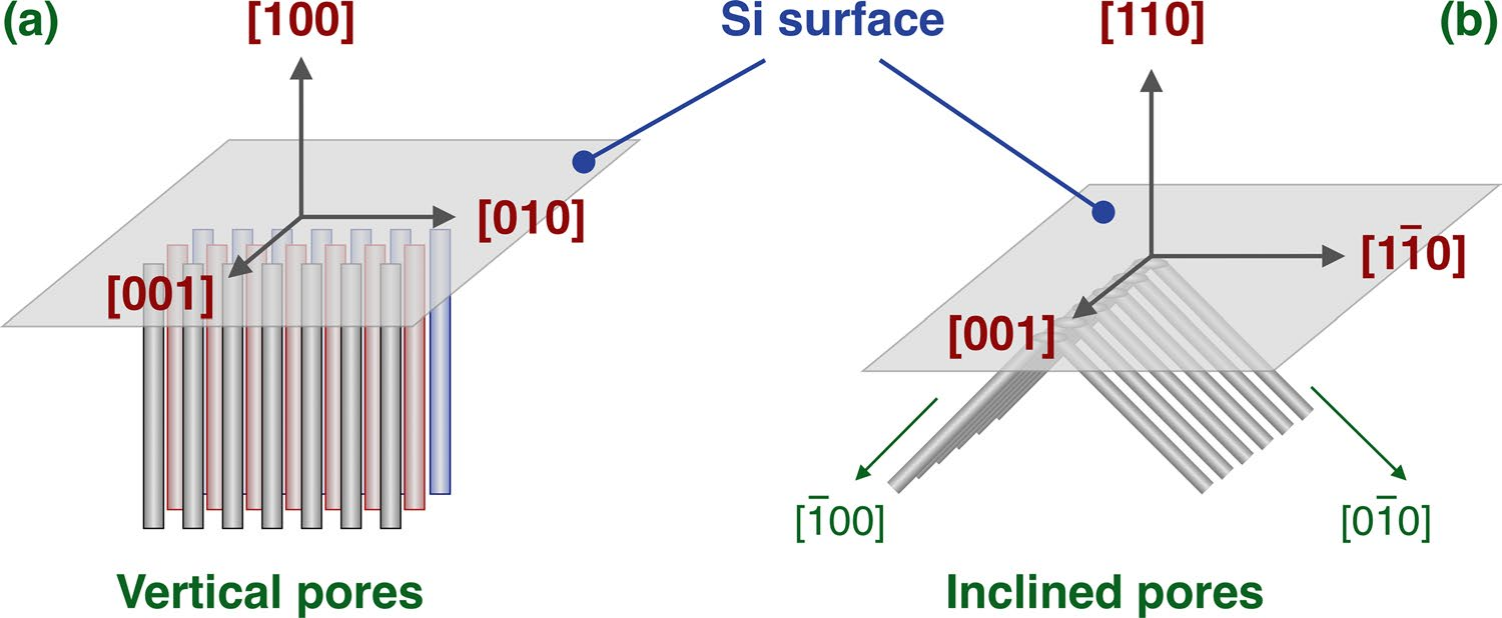
Schematic representation of PSi pores for (**a**) (100) and (**b**) (110) Si surface orientations.

**Fig. 2:**
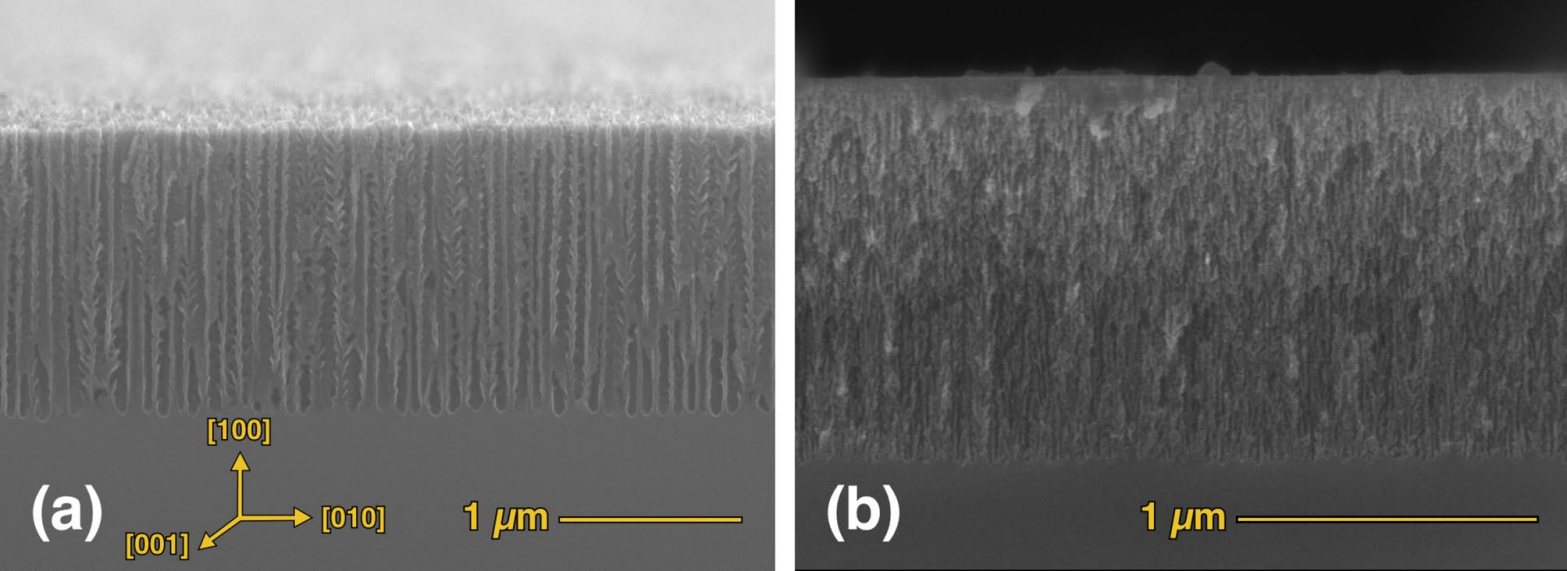
SEM cross sections of (100)-PSi layers with pore diameters of about 60 nm (**a**) and 15 nm (**b**). The crystal axes are indicated in panel (**a**) for reference.

From the point of view of the control of the structural properties, EC remains the most precise and reproducible method for a fine tailoring of the layers’ properties. For this reason, the studies on birefringence have all been made on EC-fabricated layers^42–44^. PSi pores are mainly formed along the main crystallographic axes of bulk Si^31^, namely [100], [010] and [001], and this allows to obtain dramatically different pore structures when bulk Si substrates with different crystallographic orientations are used. In Fig. 1, panels (a) and (b) show the schematic drawing of the pore structure for (100) and (110) Si surfaces, respectively. Since the pores preferential formation directions are along the main crystallographic axes, namely in the [100], [010] and [001] directions, if the EC process is started from a (100) surface then the pore structure is essentially a vertical structure, with pores formed only along [$\overline{1 }$00], since [010] and [001] directions are coplanar with the external surface and therefore are not available for pore formation (Fig. 1a). The verticality of the pore structures in (100) Si wafers can be seen in the Scanning Electron Microsopy (SEM) images in Fig. 2 of the cross sections of PSi layers with (a) 60 nm and (b) 15 nm diameter pores. Similarly, in the case of a (110) starting surface, the pores can proceed along [$\overline{1 }$00] and [0$\overline{1 }$0], since the [001] direction is along the surface and not suitable for pore formation.

The interest for the birefringence of PSi was born in the last decade of 1900s with a couple of publications that studied the optical anisotropies of p and p^+^ PSi. The first article, by F. Ferrieu et al.^42^ reports an ellipsometry study of (110)-PSi. Later in that decade, the birefringence of (100)-oriented p^+^ PSi was studied by Mihalcescu et al.^43^, where the authors investigated the effects of the structural anisotropy of (100)-PSi on the optical properties using a waveguide configuration.

### Generalities on birefringence and form birefringence

**Fig. 3:**
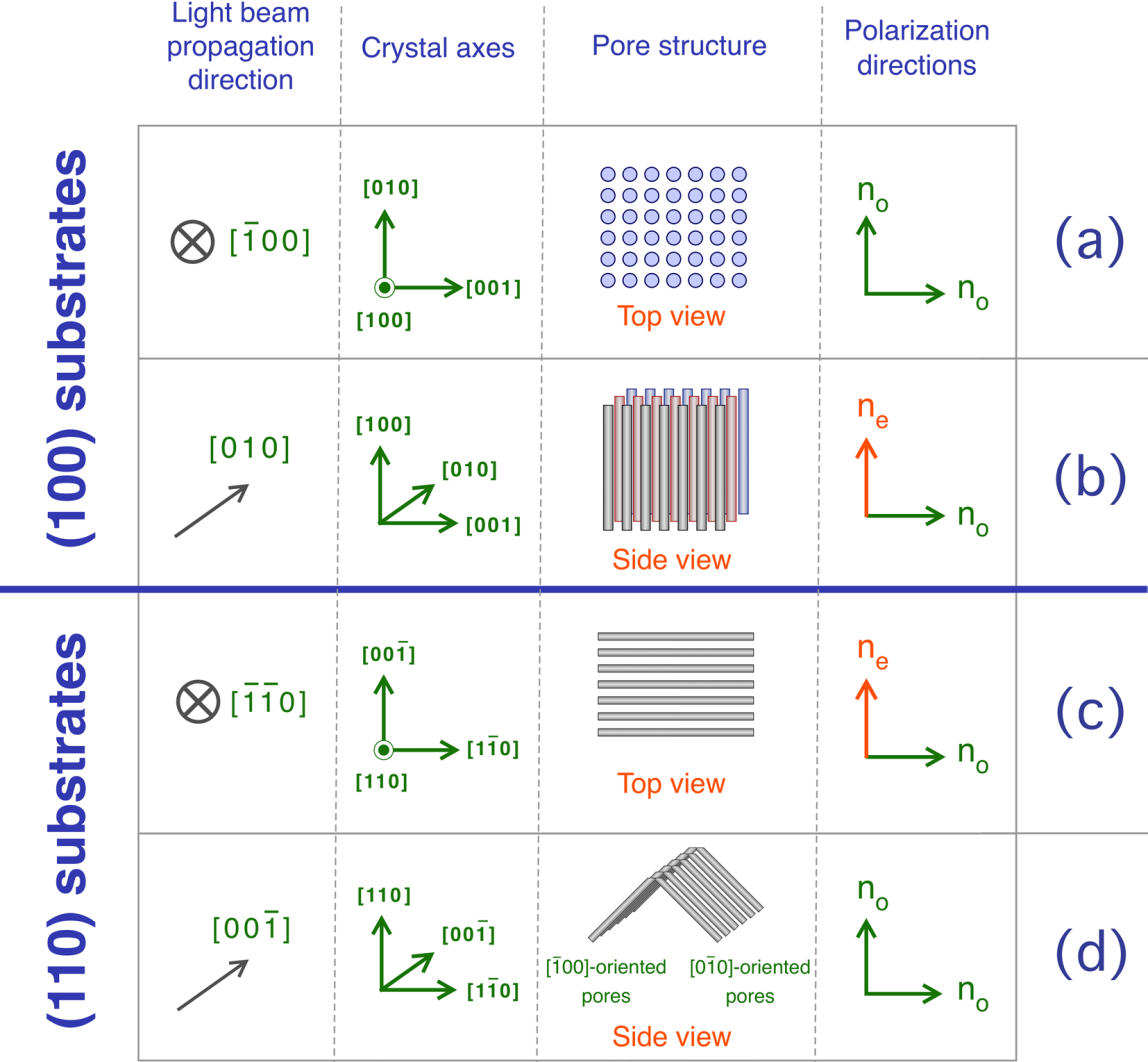
Schematic of the ordinary and extraordinary refractive indexes in a (100) and a (110) PSi layer. The crystal axes and the polarization directions with the corresponding refractive indexes are indicated. (**a**) and (**b**): top and side configurations for (100) substrates. (**c**) and (**d**): top and side configurations for (110) substrates.

For a proper discussion of our results on PSi birefringence, it is essential to explain the various terms and parameters that will be used, given that there is quite a variety of approaches in literature, where the structural and polarization approaches may significantly differ from one publication to the other. For the general description, we will refer to the book *Crystal Optics: Properties and Applications* by A.K. Bain^45^. In isotropic materials, the refractive index $n$ is independent from the light beam propagation direction *d*_*prop*_. However, in crystals where the light behavior differs along specific directions, as is the case for Iceland spar^46^, the refractive index depends on the polarization direction of the light beam, leading then to optical anisotropy. A uniaxial material exhibits two different values for the refractive index, so that along two crystal axes *n* is the same, ${n}_{1}={n}_{2}$ , and a different one, ${n}_{3}\ne {n}_{1}={n}_{2}$, is present in the third axis. We call ${n}_{1}={n}_{2}={n}_{o}$ the *ordinary refractive index* and ${n}_{3}={n}_{e}$ the *extraordinary refractive index.* If ${n}_{e}>{n}_{o}$ the material is defined as an *optically positive* birefringent material, while if ${n}_{e}<{n}_{o}$ the material is defined as an *optically negative* birefringent material. The light beam propagation direction in which the light beam experience only ${n}_{o}$, is called the *optical axis.* This polarization-dependent optical response, intrinsic to certain materials as is the case of Iceland spar, can also originate from a structural anisotropy^47–49^. This kind of birefringence, since the optical anisotropy is not given by the properties of the constituent material but depends on its spatial distribution, is referred to as “form birefringence”. The origin of the concept of “form birefringence” is attributed to F. Braun at the beginning of 1900^50^. Braun suggested that it is possible to produce an optical birefringence using a composite system built using optically isotropic constituents that are distributed on an anisotropic spatial arrangement. This is the case of PSi, where the directionality of the pores we discussed earlier generates this anisotropic spatial distribution of Si, that in its bulk form is an optically isotropic material. In the case of (100)-PSi, the object of this study, we can consider the electric field of an unpolarized light beam travelling in the [$\overline{1 }$00] direction as composed by two components, one polarized in the [010] direction and the other in the [001] direction (Fig. 3a). The optical interaction of these two polarization directions, *d*_*pol*_, with the porous layer will be the same due to the invariance of pore structure for the in-plane polarization directions, so that, in the case of (100)-oriented PSi layers, the [$\overline{1 }$00] direction is the optical axis and the refractive index experienced by the light beam is the ordinary refractive index ${n}_{o}$.

The situation significantly differs when the light travels along the [010] (or [001]) direction. In this case (Fig. 3b), the light polarized along [001] (or [010]) direction will experience a response from the material described by ${n}_{o}$, while light polarized along [100] direction will interact with a different pore geometry, so that the refractive index for this polarization will be different. We will refer to this second refractive index as the *extraordinary* refractive index ${n}_{e}$.

**Fig. 4:**
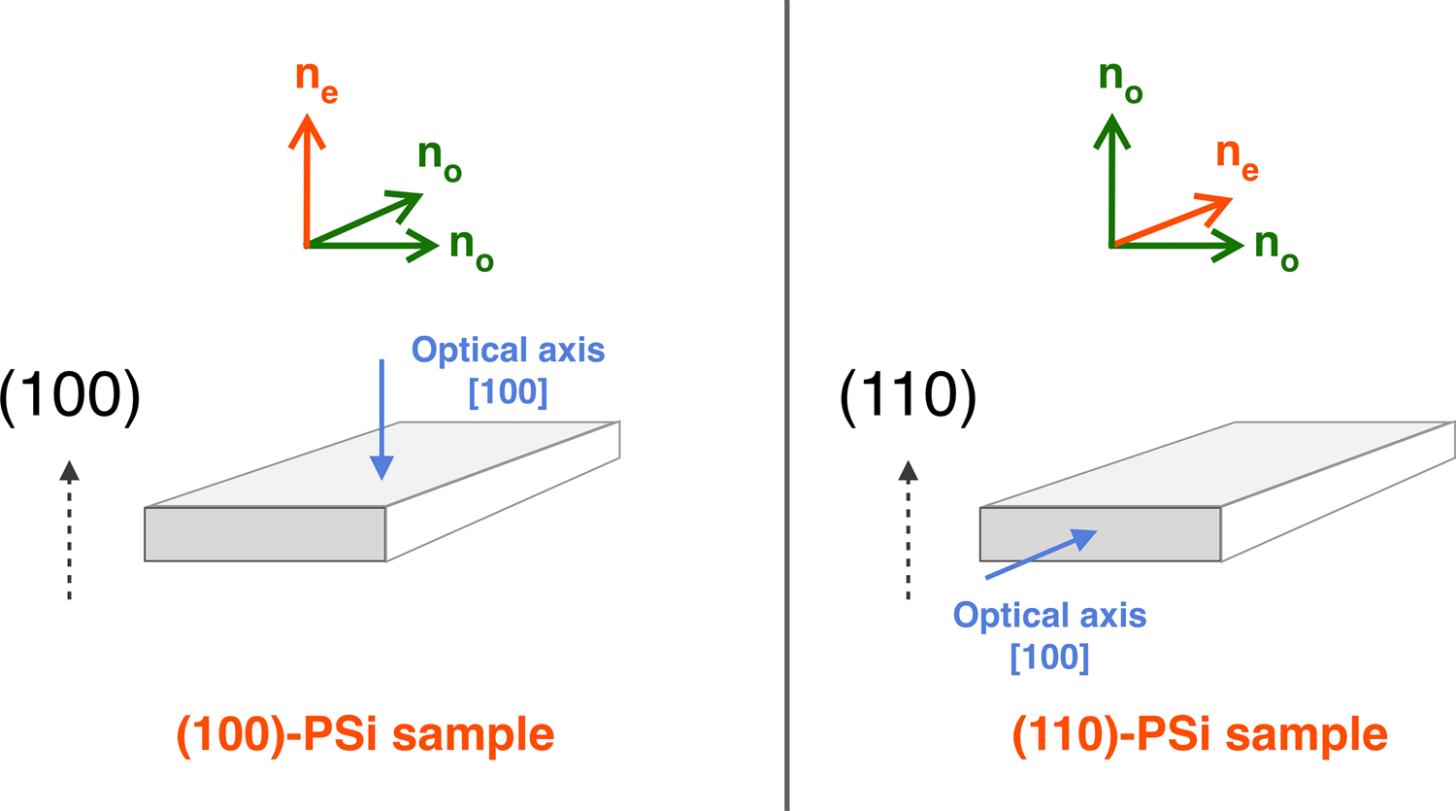
Schematic representation of the PSi optical axis when the different pore shape leads to different form birefringence configurations. The polarization directions and the refractive indexes seen by light polarized along these directions are also indicated.

The pore geometry within the porous layer changes when the top surface of bulk Si has a different crystallographic orientation, so that we can discuss what these changes produce on the optical properties of the PSi layers. Figure 3 (c) and (d) show a schematic for (110)-PSi, in analogy with the scheme for (100)-PSi in Fig. 3 (a) and (b). In this case, the main optical axis, that is the propagation direction along which light sees a homogeneous refractive index, differs from the normal to the sample’s surface. For a (110)-oriented PSi, the light beam travelling along the [100] direction will still be parallel to the optical axis, but it will be traveling along the surface and not across it, as shown in Fig. 4.

The position of the optical axis in birefringent (110)-PSi led to more studies on this crystal orientation, since it is a configuration that offer polarization-dependent values of the refractive index for a beam impinging on the surface and is therefore more suitable for the kinds of devices discussed by several authors^44,51–54^.

In this work, we will explore the birefringence properties of PSi layers formed using (100)-oriented wafers as a function of different fabrication and processing parameters and compare them with literature results in the very complex panorama of the structure-induced optical properties of PSi layers.

## Experimental details

### Porous Silicon fabrication

Porous Si layers are formed by electrochemical etching of highly doped Si wafers by using an HF-based electrochemical solution. The details of the standard fabrication methods are described in detail elsewhere^55–57^. For part of the samples, pores with diameters in the 40–80 nm range were fabricated using our patented Electrochemical NanoLithography (ENL) technique^32^ to better control the pores’ structure and distribution. We used n^++^ P-doped and p^++^ B-doped Si substrates (3–7 mΩ·cm) from Sil’tronix (France) and n^+^ P-doped Si substrates (12–15 mΩ·cm) from INSETO (UK). All wafers were (100)-oriented. The etching current density was varied in the 70–1200 mA/cm^2^, while the HF concentration was varied in the 15–35% V/V starting from a 50% HF/H_2_O RPE commercial solution and EtOH RPE, both from Carlo Erba (Italy).

The free-standing PSi membranes were prepared using an n^++^ substrate. To ensure a sufficient robustness of the membranes, we aimed at a thickness of 15 µm. Given the relatively high currents and the thickness, we opted for a pulsed formation using 8 cycles of 330 mA/cm^2^ for 15 s followed by 60 s pause (0 mA), for a total etching time of 120 s in an electrochemical solution containing 15% V/V HF. After the membrane formation, the porous layers were detached using a 5% V/V HF solution and a 111 mA/cm^2^ current density for 50 s.

### Optical characterization

Optical reflectivity measurements were made using a PerkinElmer Lambda 950 UV–Vis-NIR Spectrometer and a PerkinElmer Spectrum 100 FTIR spectrometer. The Lambda 950 spectrometer is equipped with a Universal Reflectance Accessory allowing the measurement of specular reflectivity in the 8°—63° angular range, while the Spectrum 100 spectrometer is equipped with an optical reflectivity accessory configured for measurements with an 8° incidence angle. When using the Lambda 950 spectrometer, reflectivity was measured at 8°, 20°, 40° and 60° incidence angles.

### Structural characterization

The structural characterization was performed by Scanning Electron Microscopy using a ThermoFisher FEI Inspect F™ Field Emission Gun microscope on cleaved sections of PSi layers with polarization column ranging from 20 to 5 kV and spot size 3.5, working distance 10 mm. Thickness of the porous silicon layers has been evaluated on three different points of the porous layer cross sections.

## Results and discussion

### Extraction of the optical parameters from reflectivity spectra

**Fig. 5:**
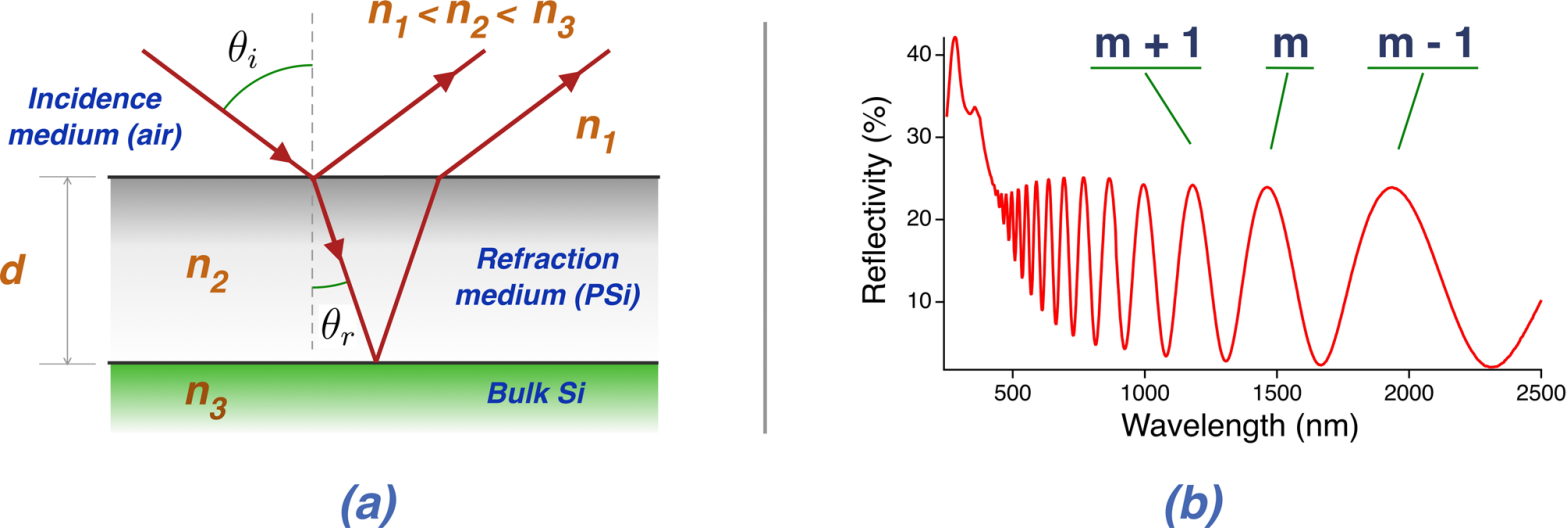
(**a**) Schematic of light refraction when light passes an interface between two media, where *n*_1_ < *n*_2_. (**b**) Typical white-light thin layer interference spectrum from a PSi thin layer. The interference order of maxima is indicated.

To be able to extract the main optical parameters, namely the thickness *d* and the real part of the effective refractive index *n*, from our reflectivity measurements, we fabricated our samples as thin layers from which thin-layer interference maxima can be seen. The experimental scheme with the indication of the parameters involved in the refraction phenomena is shown in Fig. 5(a), while an example of reflectivity spectrum from a thin PSi film is shown in Fig. 5(b). The interference fringes are clearly visible in the spectrum.

By combining the Bragg’s law and the Snell’s law we can retrieve *d* and *n* from the reflectivity spectra collected at different angles (see details in Supplementary Materials). Following the analysis in Supplementary Materials, the refractive index ${n}_{2}$ can be obtained as the fitting parameter of the following expression:

1 $$2n_{2} d cos(\sin^{ - 1} \left( {\sin \theta_{i} /n_{2} )} \right) = m\lambda_{{\theta_{i} }}$$

### PSi layers birefringence

The birefringence of (100)-PSi layers can be studied by analyzing the reflectivity spectra for different ${\theta }_{i}$. Since we are dealing with a birefringent material, the layer’s refractive index that will be measured, indicated as ${n}_{2}$ in Fig. 5, will be an effective refractive index *n*_*eff*_ that will come from a combination of ${n}_{o}$ and ${n}_{e}$ that will depend on the orientation of the incident light beam. For light impinging along the optical axis *n*_*eff*_ = ${n}_{o}$.

**Fig. 6:**
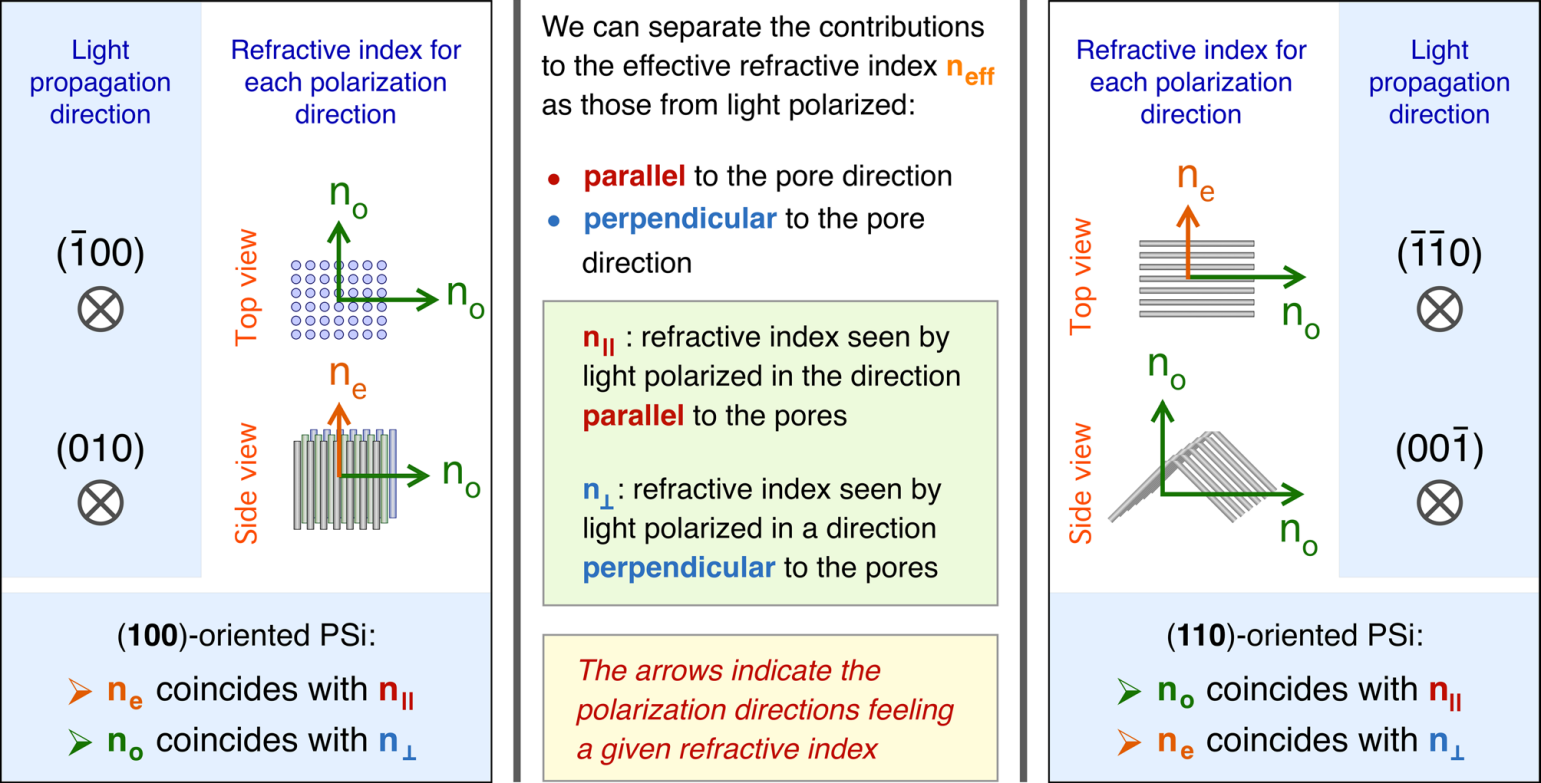
Identification of the contribution to the effective refractive index n_eff_ from the polarization directions within the porous structure. The incident light propagation directions are also indicated within each panel. Left: (100)-PSi layers. Right: (110)-PSi layers.

Given the pores orientation within the porous layers as a function of the Si wafer surface orientation, we can separate the contributions to *n*_*eff*_ given by the different light polarization directions, in particular if light is polarized along or across the pores (Fig. 6). First, we can analyze the case of (100)-oriented substrates (Fig. 6, left panel) whose optical axis is the [100] direction (see Fig. 4 for reference). A light beam propagating along the optical axis will have polarization directions parallel to the surface, so that the ordinary refractive index ${n}_{o}$ is, in this configuration, the one seen by the light polarized *perpendicularly* to the pores. We can call this component ${n}_{\perp }={n}_{o}$. When the light is coming laterally, say for instance along [010] direction, unpolarized light will have a component polarized across the pores that will see ${n}_{\perp }$ again and a component polarized *parallel* to the pores that will see a different material structure and will therefore experience a different interaction with the porous layer and a different refractive index, that we will call ${n}_{\parallel }$. For the geometrical configuration just described, in the case of (100)-PSi the extraordinary refractive index ${n}_{e}$ is the one seen by the light polarized parallel to the pore direction and, therefore ${n}_{\parallel }={n}_{e}$. This situation is summarized in Eq. (2) in the case of a negative birefringent (100)-oriented PSi:

2 $$\left( {100} \right) \to n_{e} = n_{\parallel } < n_{o} = n_{ \bot }$$

In the case of (110)-oriented substrates (Fig. 6, right panel), the optical axis is still along the [100] direction, that in this case, as discussed earlier, it is not perpendicular to the surface but parallel to it. The light propagating along the optical axis will have polarization directions parallel to the pores, so that in this case we have ${n}_{\mathrm{o}}$_o_ $={n}_{\parallel }$. When light arrives along the normal to the surface, the [110] direction, the light interaction with the porous layer will depend on the polarization direction (see Fig. 6, top right). Light polarized parallel to the pores will see the same refractive index as the light propagating along the optical axis, that is ${n}_{\mathrm{o}}={n}_{\parallel }$. However, the light polarized across the pores will see the extraordinary refractive index ${n}_{\mathrm{e}}={n}_{\perp }$. This is summarized in Eq. (3), again for a negative birefringent material:

3 $$\left( {110} \right) \to n_{o} = n_{\parallel } > n_{e} = n_{ \bot }$$

When comparing Eq. (2) and Eq. (3), it becomes apparent that to have a negative birefringent material for both orientations ${n}_{\parallel }$ must be smaller than ${n}_{\perp }$ in one case (the (100) orientation) and larger in the other (the (110) orientation). Since the literature reports (110)-PSi layers as a negative birefringent material^44,51,52,54,58,59^ we should expect that (100)-PSi layers should be positive birefringent materials, as has been reported for p- and p^++^-type (100)-PSi^43,58^

**Fig. 7:**
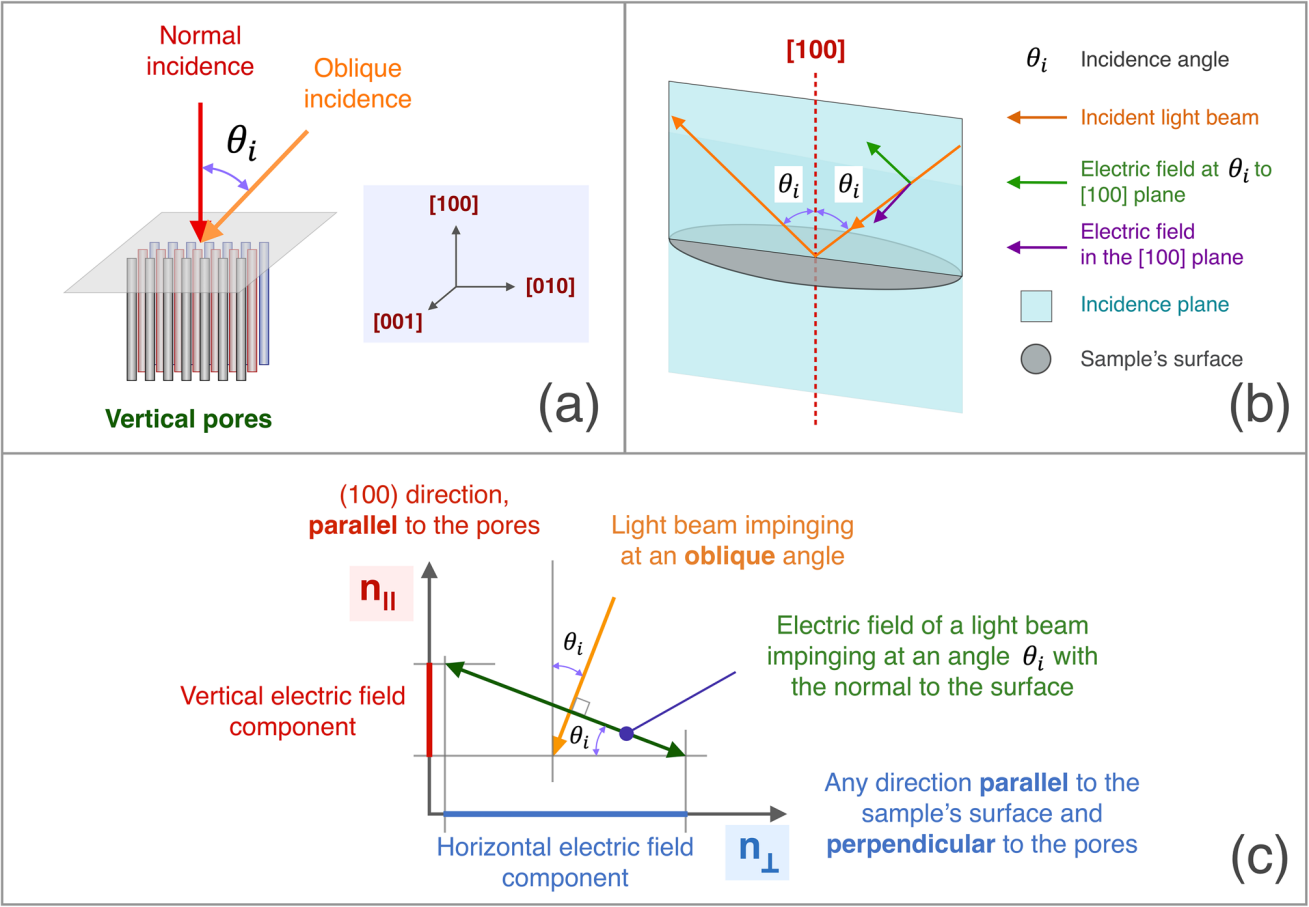
Schematic of the different contributions from n_o_ and n_e_ within our measurements with unpolarized or partially polarized light. (**a**) Schematic of light beams with normal ($\approx 0^\circ$) and oblique (${\theta }_{i}$) incidence. (**b**) When in oblique incidence, the understanding of the different contributions to the optical properties comes from separating the polarization direction parallel to the [100] plane from the one at a θi incidence with respect to that plane. (c) For oblique incidence, the electric field for the off-normal polarization can be separated in a component along the pores (${n}_{\parallel }$) and one perpendicular to them and parallel to the [100] plane (${n}_{\perp }$).

Figure 7 describes what happens in our configuration, that is for (100)-oriented PSi. We will consider two possible cases (Fig. 7a): light impinging perpendicularly to the sample’s surface and at a given angle ${\theta }_{i}$. In the first case, the light beam will see just the ordinary refractive index ${n}_{o}={n}_{\perp }$. At non normal incidence, the light will see an effective refractive index *n*_*eff*_ that will be a combination of ${n}_{\parallel }$ and ${n}_{\perp }$ since the two polarization directions (perpendicular and inclined with respect to the optical axis) will see those two different refractive indexes (Fig. 7b). At a given ${\theta }_{i}$, the component of the electric field at ${\theta }_{i}$ with the optical axis can further be divided in two more components, one vertical, along the optical axis, and one horizontal, perpendicular to it (Fig. 7c). Based on these considerations, we can see that contribution of ${n}_{e}={n}_{\parallel }$ to *n*_*eff*_ will depend on ${\theta }_{i}$ and will be increasing with increasing ${\theta }_{i}$. If ${n}_{e}$ > ${n}_{o}$ we’ll observe an increase of *n*_*eff*_ while increasing ${\theta }_{i}$ and the PSi layer will be an *optically positive* birefringent material. On the other side, if ${n}_{e}$ < ${n}_{o}$ we will observe a decrease of *n*_*eff*_ and the PSi layer will behave as an *optically negative* birefringent material^31^.

**Fig. 8:**
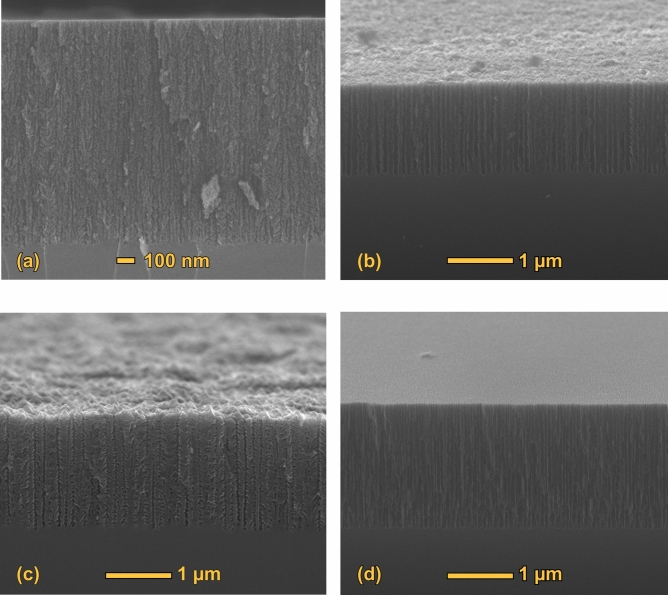
SEM cross-section images of a typical set of PSi samples used in this study. Panels (**a**) and (**b**) shows two different pore shapes for similar porosities in the 40–60% range, showing the control that is separately possible over porosity and pore shape. Panel (**c**) show a low porosity ($\sim$ 35%) sample while panel (**d**) shows a high porosity ($\sim$ 80%) sample. Porosity is defined as the ratio of empty over total volume of a given sample. The fabrication parameters for these samples are: (**a**) 45 mA/cm^2^, 15% HF, 25 s fabrication time, n^++^ substrate; (**b**) 1200 mA/cm^2^, 22% HF, 2 s fabrication time, n^+^ substrate; (**c**) 700 mA/cm^2^, 22% HF, 4 s fabrication time, n^+^ substrate; (**d**) 330 mA/cm^2^, 15% HF, 15 s fabrication time, n^++^ substrate.

In Fig. 8 we show four cross-section images of typical (100) PSi samples used in this study. The porosities are measured by gravimetry^31^. Panels (a) and (b) show two samples having a different pore structure while having a similar porosity, showing the separate control given by electrochemical parameters over pore shape and sample’s porosity. Panels (c) and (d) show two samples with low and high porosities, respectively. We used SEM to image a large number of samples (several tens) in cross-section to determine the intrinsic variability of the EC fabrication process, verifying its high reproducibility and the excellent predictability of samples’ thickness due to its reliable dependence on the EC parameters and process duration. As a rule, our results are in full agreement with the high reproducibility of the EC fabrication process well known in the scientific literature^31^.

**Fig. 9:**
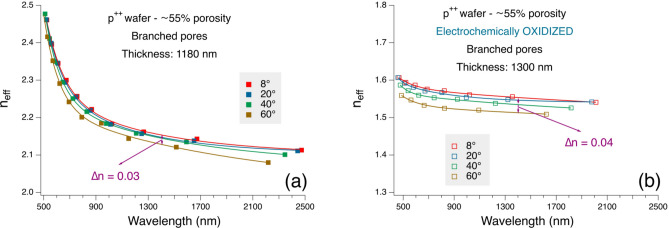
Spectral evolution of n_eff_ for a (100)-oriented p^++^-type PSi sample for four different incidence angles. The full lines are added as guide for eyes. (**a**) as prepared; (**b**) after electrochemical oxidation.

Figure 9 shows the evolution of *n*_*eff*_ in a p^++^ sample prepared using a 30% HF ethanoic solution and a 75 mA/cm^2^ formation current for 30,0 s as a function of the wavelength. The thickness and *n*_*eff*_ values are calculated using the methodology described the Supplementary Materials. Each experimental point corresponds to the value for a given interference maximum. For consistency, we also checked the correspondence of the layer thicknesses measured by SEM and the one calculated using Eq. (1), and found an excellent agreement, especially for 40%-60% porosities. For instance, for samples in this porosity range, the difference between the SEM thickness and the one we determined using our calculations from reflectivity spectra disagree only for a couple of tens of nm over thicknesses of 1400 nm (e.g. 1400 by SEM and 1430 by reflectivity).

The curves in Fig. 9 show the evolution of the real part of the measured refractive index *n*_*eff*_ for four incidence angles, namely 8°, 20°, 40° and 60°. Although the model has its main validity in the below-the-gap spectral region, we also plotted the measured refractive index up to the green spectral range. The observed behavior is regular and is consistent with the values expected for a 55% porosity. The spectral evolution, showing an increase for shorter wavelengths is also consistent with the literature results^60^.

The results in Fig. 9(a) demonstrate that in our case the samples show a negative birefringence. Please note that the difference shown is among the values of *n*_*eff*_ at different incidence angles and not directly $\Delta n={n}_{\mathrm{e}}-{n}_{\mathrm{o}}$. This implies that the real $\Delta n$ is larger than what we can directly measure with the spectrometer, as previously discussed.

As a further step to understand the samples’ birefringence, we electrochemically oxidized the sample in Fig. 9(a) using a 0,1 M KNO_3_ solution in water and a constant current of 4 mA/cm^2^ until the instrumental upper limit for the applied voltage (10 V) has been reached ($\sim$ 1 ks). Please note that electrochemical oxidation of PSi layers cannot lead to a full oxidation of the Si skeleton, since it starts from the developed surface in contact with the oxidation solution and has its end when, in the constant current configuration, the instruments reach the applied voltage limit, that in our case is 10 V. Although we did not measure the oxygen concentration in this study, based on previous results on PSi samples with similar structure properties^55^, we can evaluate the oxygen concentration in the sample in Fig. 9(b) as about 50%, meaning that only a quarter of the available Si atoms is fully oxidized if we consider only SiO_2_ formation. This leaves the possibility that the surface modifications affect the electrical conductivity of the porous layer^61^ that can, in turn, affect the observed birefringent behavior. Please note that the different in-plane and vertical conductivities are likely a relevant parameter in the observed behavior, since they directly affect the interaction of the light with the structure for the different polarization. The results in Fig. 9(b) show a decrease of the overall refractive index and a flattening of the spectra related to the formation of the SiO_2_, but also a slight increase of the birefringence.

**Fig. 10:**
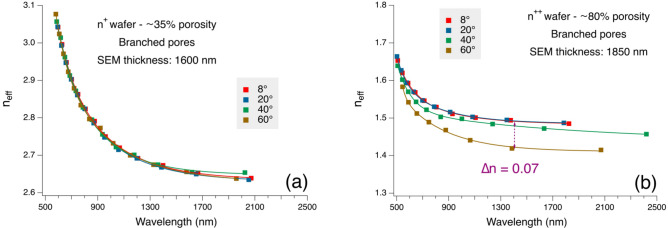
Spectral evolution of n_eff_ for (**a**) a low porosity and (**b**) a high porosity (100)-oriented n^+^-type PSi samples for four different incidence angles. The full lines are added as guide for eyes.

To explore the effect of the porosity of our highly doped samples on the observed birefringence we report in Fig. 10 (a) and (b) the spectral evolution of *n*_*eff*_ for a low- and a high-porosity samples, respectively. The low porosity sample has been prepared on an n^+^ Si substrate using 700 mA/cm^2^, 22% HF, 4 s fabrication time, while the high porosity sample has been prepared on a n^++^ Si substrate using 330 mA/cm^2^ in a 15% HF solution for 15 s. The low porosity sample shows a negligible birefringence, while the Δ*n* observed in the high-porosity sample is significantly higher. These results are consistent with those reported in Fig. 9 and show how increasing the anisotropy increases the form birefringence in PSi.

### PSi membrane for polarization transformation

**Fig. 11:**
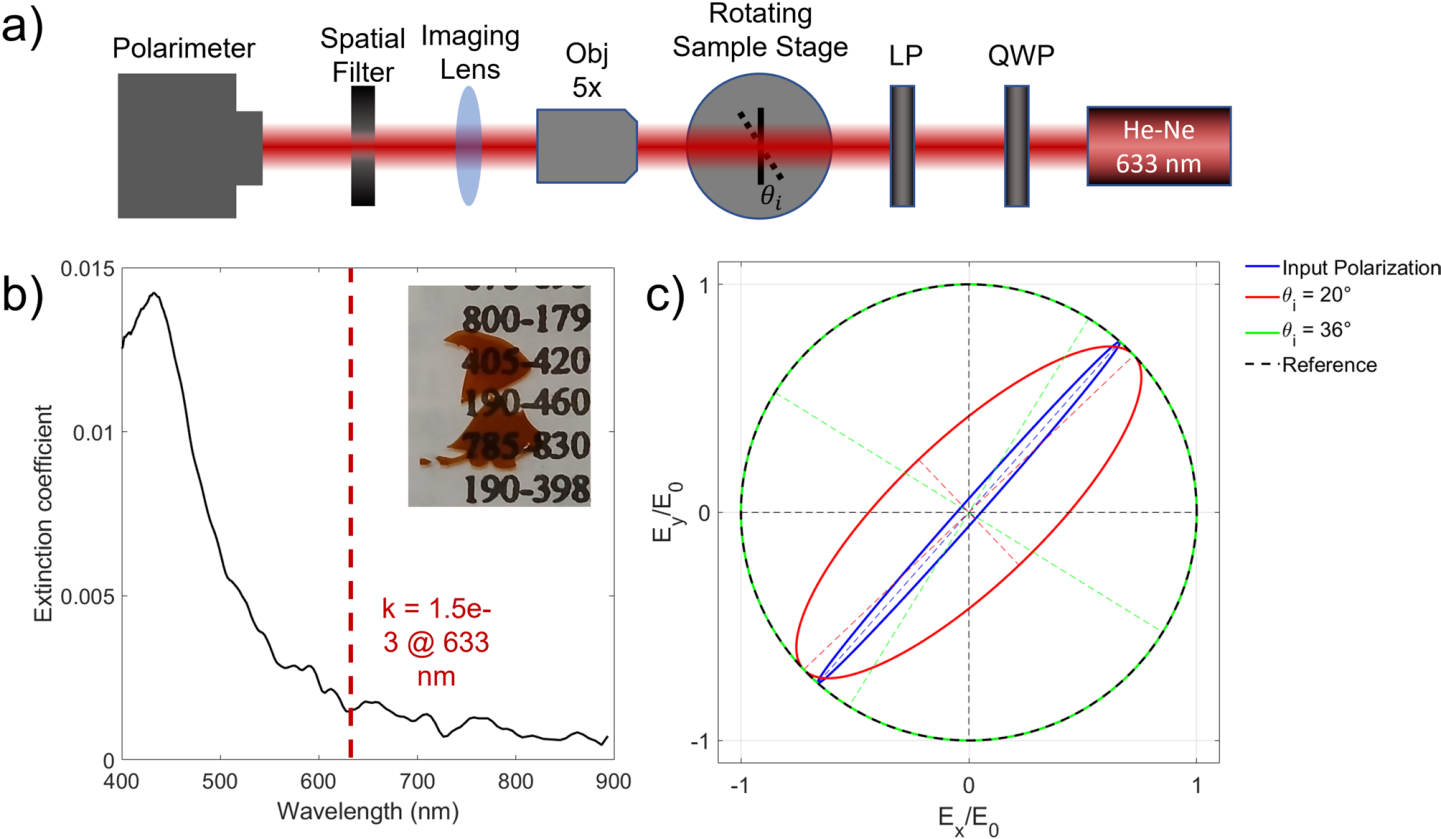
(**a**). Schematic view of the optical setup for the polarimetric characterization of the PSi membrane. (**b**). Measured extinction coefficient of the membrane. The picture of the PSi membrane is shown in the inset (**c**). Ellipses of polarization measured at different incidence angles ${\theta }_{i}$.

The optical birefringence exhibited by (100)-oriented samples can be exploited to manipulate the polarization state of light passing through the material. To this aim, we fabricated PSi membranes 15 µm thick (see Experimental details section) and mounted them on a rotating stage within the optical setup sketched in Fig. 11 (a). Figure 11 (b) shows the extinction coefficient as a function of the wavelength, while the image in the inset shows the transparency of the PSi membrane in the red part of the spectrum. We therefore make use of a linearly polarized He–Ne laser (633 nm) to assess the ability of the membrane to change its polarization state.

Figure 11 (c) shows the ellipse of polarization when the sample rotates around the y-axis. At normal incidence (${\theta }_{i}$ = 0°) the membrane has no effect, and the laser is linearly polarized at about 45° in the xy-plane (blue ellipse). By rotating the membrane, the ellipticity increases (red ellipse, ${\theta }_{i}$ = 20°) and circularly polarized light is obtained when ${\theta }_{i}$ = 36° (green ellipse). In this configuration, the membrane acts as a quarter waveplate with a Degree of Polarization (DOP) as high as 98%. The parameters of the polarimetric characterization are reported in Table 1.

**Table 1:** Polarimetric parameters measured at different incidence angles.

${\theta }_{i}$	S_1_ norm	S_2_ norm	S_3_ norm	$\alpha$(azimuth)	DOP	$\Delta \varphi$
0° (input)	− 0.12	0.99	− 0.08	48.32	96.83%	− 4.63
20°	0.04	0.81	− 0.58	43.43	98.27%	− 35.84
36°	0.01	− 0.02	− 1	− 27.68*	98%	− 91.07

### Discussion

Before discussing the results, relevant light diffusion/scattering effects in our measurements that could be generated by the nanoscale structure of our samples have to be excluded. To this aim, the first reason to exclude these effects is that our analysis is mainly related to the transparency region of PSi, that is essentially for wavelengths longer than about 800 nm and up to 2500 nm. Given that the diameter of our pores is 40-80 nm, this means that the Mie scattering (relevant for particle sizes larger than the 1/10 of the impinging light wavelength) is negligible. Only Rayleigh scattering remains and, based on literature results on similar material, it also has negligible impact on samples having our pore size, thickness and refractive index^62,63^. Moreover, the scattering intensity depends on the thickness of the samples^62^, so that, again, we can neglect possible light scattering effects in this study.

All our samples behave as negative birefringent materials. Although this seems in contrast with the previous results from various authors in literature, such a discrepancy is not that surprising considering the variety of parameters involved. For instance, Künzer et al.^44^ in their 2005 work noted that the birefringent behavior strongly depends on the doping level of their samples, ranging from positive to negative birefringence and the porosity itself affects the birefringent behavior. The very high sensitivity of the optical birefringence properties on the details of the PSi structure is such that (110)-oriented layers, although generally considered uniaxial materials, are in reality biaxial structures with three different refractive indexes ${n}_{[001]}$, ${n}_{[1\overline{1 }0]}$ and ${n}_{[110]}$, as shown in the work by Shichi et al.^59^.

The doping kind is also a relevant parameter to be considered, and n- and p-type samples having the same resistivity can show remarkably different properties. As an example, a different Young’s modulus has been reported for p- and n-type PSi^64,65^. The strain-related effects^66^ in PSi layers have already been shown to affect the complex refractive index^67^ and even other properties such as the adsorption of molecules onto the PSi inner surface^68^. The significant differences in the electrochemical behavior for different kinds of doping^31^ are also a strong indication that the different free charges behavior strongly affects the overall properties of these mesoporous layers. An interesting paper on this aspect is the work by Melhem et al.^69^. Even the fabrication procedures for free-standing PSi layers significantly differ: for p^+^-type a relatively high current density (several hundreds of mA/cm^2^) using solutions with a relatively high (10–22%) HF concentration is needed^70,71^, while for n^+^-type the only viable configuration we found was a 5% HF etching solution and about 100 mA/cm^2^ current density (see Experimental details).

It is very complex to look for the specific physical origin of positive or negative birefringence in PSi that would also explain the surprising, although small, increase of the birefringence after the electrochemical oxidation. The linearity and simplicity of our approach are a strong support to the soundness of our experimental findings, but the intrinsic physical origin of the observed behavior remains unclear. A brief excursus in the literature models shows that the interpretations are all based on averaged approximations. For instance, Golovan et al.^54^ use the Bruggeman effective medium approximation considering Si nanocluster as ellipsoids to model their results and fit them. The same approach is used by J. Àlvares et al. in their work^72^. A different choice has been opted for by Mihalcescu et al.^43^, that used the Maxwell–Garnett formula and what they define an “oversimplified model” depicting the (100) PSi structure as cylinders. Within this model, they attribute the observed birefringence to a morphological anisotropy thanks to a rough general agreement between their model and the experimental data. Künzner et al.^44^ use again the Bruggeman effective medium approximation and ellipsoids and, consistently with the other authors, attribute the observed birefringence to a structural anisotropy of PSi and conclude that (110)-PSi is a uniaxial material. They also however observe a reversed optical birefringence for p^++^ and p^+^ layers even if they conclude that they are both negative uniaxial materials. Please note that while Golovan et al., Künzner et al. and J. Álvarez et al. use the Bruggeman model for porosities above the 30%, Mihalcescu et al. justify their choice of the Maxwell–Garnett formula by suggesting that the Bruggeman model is not the best suited for porosities higher than 30%. All these studies have one thing in common: they do not go further than attributing the observed birefringence to a structural anisotropy of the porous layer thanks to an effective medium approximation that is also non homogeneously recognized in literature as the best suited for all porosities. Moreover, the approach used being an average model, it is intrinsically unable to take into account the impact of the many parameters we cited above, or effects as electron mobility in p^++^ or n^++^ PSi samples, as it only uses the standard Si dielectric constant. This implies that the physical origin of the birefringence being positive or negative in PSi has never been really investigated from the theoretical point of view. Keeping in mind the research results of Shichi et al^59^, where they conclude that “the structure of (110) PSi is more complex than expected” since it is a biaxial and not an uniaxial material, and that “to perform realistic calculations” this complexity has to be taken into account, it is apparent that a computational approach for a thorough understanding of PSi form birefringence in its various crystal orientations is far from being obvious. Given that the standard approach for the PSi birefringence does not and cannot explain our results, a whole new approach has to be developed and new experimental information, that will be the object of future works, has to be collected.

The relevant impact of all these parameters on the optical properties of PSi layers, despite the complexity of the general picture and the difficulty of identifying an actual physical model, offers however a remarkable range of prospective control tools, a potential that makes PSi a very versatile tool aimed at optical sensors for every application where the detection is related to structural modifications as pore filling or pore surface changes. For instance, Künzner et al.^44^ showed how pore filling affects the birefringent properties of the porous layer, S. Álvarez et al. explicitly studied the potential use of form birefringent PSi for optical sensing^72^, and similar devices have been explored with other materials as porous alumina^73^. Our results further demonstrate that proper design of the PSi structure can lead to high sensitivity devices where the birefringence plays a significant role instead of standard methods involving reflectivity or luminescence.

## Conclusions

To summarize our results, we have shown that a simple characterization method can be derived from Bragg and Snell laws that allow the determination of the optical parameters of thin layers. In the case of PSi, we have analyzed the behavior of (100)-oriented samples with different doping levels, all in the high or very high doping level range. The optical determination of the thickness was in excellent agreement with the SEM microscopy measurements, especially for porosities in the 40%—60% range. Above or below this range, it is possible that the reduced contrast with, respectively, air or bulk Si can explain why the methodology is in good but less precise agreement with the microscopy measurements. Using SEM or optically determined thickness, our samples all show a negative birefringence behavior, whose intensity increases with the porosity and show no attenuation with electrochemical oxidation. Further work is in progress to discern what parameters affect most the birefringent behavior and how to accurately control their effect to tailor the samples’ performances.

## Supplementary Information

Supplementary Information.

## Data Availability

All data needed to evaluate the conclusions in the paper are present in the paper. Additional data related to this paper may be requested from the authors.

## Abbreviations

ENL: Electrochemical NanoLithography
PSI: Porous silicon
SEM: Scanning electron microscopy
