# Supplementary material for: Dramatic changes induced on porous silicon birefringence by shape-dependent properties
Source: Sci Rep. 2026 Mar 30;16:15198. doi: 10.1038/s41598-026-41405-6 (PMC13183964; doi:10.1038/s41598-026-41405-6)
Supplement: Supplementary file 1 — Supplementary Information. [file 41598_2026_41405_MOESM1_ESM.pdf]

Supplementary Materials

Dramatic changes induced on Porous Silicon birefringence by shape-dependent properties

Guido Mula[1,], Muhammad Naseem Akhtar[1], Francesca Assunta Pisu[1], Stéphane Bastide[2], Angelo Angelini[3,*], Mateo Rosero-Realpe[4], Luca Boarino[3]*

[1] PoroSiLab, Dipartimento di Fisica, Università degli Studi di Cagliari, Cittadella Universitaria di Monserrato, S.P. 8 km 0.700, Monserrato (Ca), Italy

[2] Univ Paris Est Créteil, CNRS, ICMPE, UMR 7182, 2 rue Henri Dunant, Thiais 94320, France

[3] Advanced Materials & Life Sciences Division, Istituto Nazionale di Ricerca Metrologica,

Optical constants retrieval method

We can use the reflectivity spectra at different incidence angles θ_i to experimentally determine the values of the layer thickness *d* and of its refractive index *n*. Let us start with the Bragg conditions for thin layer interference for a thin PSi layer:

$$2nd\, cos\, \theta_r = m\, \lambda$$

where θ_r is the refraction angle within the thin layer. To obtain the value of $2nd$ we can approximate $\cos\theta_r$ to 1 if the incidence angle θ_i is small enough to assimilate that angle to normal incidence. For $\theta_i = 8°$ we obtain $\cos 8° = 0{,}99$ that is a 1% difference. Since in our configuration the incidence medium is air and therefore $n_{\mathrm{air}} < n_{\mathrm{PSi}}$, for any given incidence angle θ_i we have $\theta_r < \theta_i$, so that that we can assume 1% as the maximum error induced by this approximation.

Using this approximation, we can calculate the value of the refraction angle θ_r by measuring the optical reflectivity at $\theta_i = 8°$ and $\theta_i = 20°$ and dividing Eq. (1) for $\theta_i = 20°$ by its expression for $\theta_i = 8°$. For a given interference maxima ordinal number *m*, the same at the two incidence angles, we obtain:

$$\cos\theta_r^{20°} = (m\,\lambda_{20°})/(m\,\lambda_{8°}) = \lambda_{20°}/\lambda_{8°}$$

where $\lambda_{20°}$ and $\lambda_{8°}$ are the wavelength at which the mth maximum occurs when the incidence angle is 20° and 8°, respectively, while $\theta_r^{20°}$ is the refraction angle for 20° incidence. With this information, the refraction angle $\theta_r^{20°}$ is given by:

$$\theta_r^{20°} = \cos^{-1}(\lambda_{20°}/\lambda_{8°})$$

We can also express the Snell law for $\theta_i = 20°$:

$$n_1 \sin 20° = n_2 \sin\theta_r^{20°} = \sin(\cos^{-1}(\lambda_{20°}/\lambda_{8°}))$$

where n_1 and n_2 are the refractive indexes of the incidence and refraction media, respectively, to express n_2 as a function of known or measured parameters:

$$n_2 = sin\,20°/sin\,\theta_r^{20°}$$

We can now calculate the value of the thickness *d* of our sample as:

$$d = (m\,\lambda_{8°})/(2\,n_2) = \frac{1}{2}\cdot m\,\lambda_{8°}\cdot\left(\frac{sin\,(20°)}{sin\,(\theta_r^{20°})}\right)^{-1} \qquad (1)$$

Since in this expression all values are known, with the only approximation of considering $\cos(\theta_r) \cong 1$ for $\theta_i = 8°$ we can calculate a reliable value of *d*. The limit of this calculation is that its application is optimal for spectral regions where the refractive index is expected not to change

significantly in the range related to the interference fringes displacements when the incidence angle is varied from 8° to 20°. We can of course reduce the variation of θ_i if needed, but measuring d for wavelengths larger that 1400 nm is sufficient for the aim of obtaining a reliable d since for PSi the refractive index in that region has a very slow variation[74]. It is worth noting that staying in the infrared part of the spectrum has also the advantage that the imaginary part of the refractive index is zero since it is below the Si indirect gap and therefore there is no absorption. This is important since in our discussion we are dealing only with the real part of the refractive index.

To avoid any further approximation, we'll use the value of d we just derived to calculate the value of n_2 as a fitting parameter in the following expression, that is a straightforward combination of the Bragg and Snell laws when the incidence medium is air or vacuum (n_1 = 1):

$$2n_2 d\, cos(sin^{-1}(sin\,\theta_i/n_2)) = m\lambda_{\theta_i} \tag{2}$$

All parameters in Eq. (2) are known except n_2, so that n_2 can be obtained as the value that validates the identity. With this method, we can obtain the value of the real part of the refractive index at different wavelengths, using the interference maxima, and for different θ_i.
